# Boosted Harris Hawks Shuffled Shepherd Optimization Augmented Deep Learning based motor imagery classification for brain computer interface

**DOI:** 10.1371/journal.pone.0313261

**Published:** 2024-11-21

**Authors:** Fatmah Yousef Assiri, Mahmoud Ragab

**Affiliations:** 1 Software Engineering Department, College of Computer Science and Engineering, University of Jeddah, Jeddah, Saudi Arabia; 2 Information Technology Department, Faculty of Computing and Information Technology, King Abdulaziz University, Jeddah, Saudi Arabia; University 20 Aout 1955 skikda, Algeria, ALGERIA

## Abstract

Motor imagery (MI) classification has been commonly employed in making brain-computer interfaces (BCI) to manage the outside tools as a substitute neural muscular path. Effectual MI classification in BCI improves communication and mobility for people with a breakdown or motor damage, delivering a bridge between the brain’s intentions and exterior actions. Employing electroencephalography (EEG) or aggressive neural recordings, machine learning (ML) methods are used to interpret patterns of brain action linked with motor image tasks. These models frequently depend upon models like support vector machine (SVM) or deep learning (DL) to distinguish among dissimilar MI classes, such as visualizing left or right limb actions. This procedure allows individuals, particularly those with motor disabilities, to utilize their opinions to command exterior devices like robotic limbs or computer borders. This article presents a Boosted Harris Hawks Shuffled Shepherd Optimization Augmented Deep Learning (BHHSHO-DL) technique based on Motor Imagery Classification for BCI. The BHHSHO-DL technique mainly exploits the hyperparameter-tuned DL approach for MI identification for BCI. Initially, the BHHSHO-DL technique performs data preprocessing utilizing the wavelet packet decomposition (WPD) model. Besides, the enhanced densely connected networks (DenseNet) model extracts the preprocessed data’s complex and hierarchical feature patterns. Meanwhile, the BHHSHO technique-based hyperparameter tuning process is accomplished to elect optimal parameter values of the enhanced DenseNet model. Finally, the classification procedure is implemented by utilizing the convolutional autoencoder (CAE) model. The simulation value of the BHHSHO-DL methodology is performed on a benchmark dataset. The performance validation of the BHHSHO-DL methodology portrayed a superior accuracy value of 98.15% and 92.23% over other techniques under BCIC-III and BCIC-IV datasets.

## 1. Introduction

BCI is a developing technique that can determine brain activities and transform them into simulated outcomes such as supplementing, restoring, improving, replacing, or enhancing natural CNS outcomes [1]. In various BCI tasks, MI is a prevalent model and is usually described as imagining the movements of a body part without real motor implementation. It will be demonstrated for sharing the same mechanism as real motor execution and allow human focus to control a robotic arm, drone, and computer cursor [2]. It can support patients with motor disabilities’ interaction with their surroundings by controlling devices like wheelchairs, prostheses, and computer cursors. EEG-based BCI is a category of commonly employed BCI methods because of its non-invasive nature, which does not require some surgical technique [3]. Among the significant modules of these BCI systems is the classification of restricted and transient EEG modifications such as event-related synchronization (ERS) or event-related desynchronization (ERD) in diverse categories of MIs [4]. A wide-ranging EEG-based BCI model comprises four parts: pattern classification, signal preprocessing, EEG signal acquisition, and feature extraction [5]. In the primary measure, feature extraction is described as employing different frequency, time, or spatial domain processing methods for extracting the features, which will be executed for classification, and decreasing the dimensions of feature vectors to satisfy the real-time process requisites [6].

The existing MIs EEG-based BCI scheme mainly employs traditional signal processing or ML techniques for feature extractors and classification. Artificial intelligence (AI) is defined as the computers or systems that emulate human intelligence to carry out tasks and will (iteratively) enhance themselves depending on the data that was obtained [7]. AI will provide numerous forms comprising ML and DL. ML has been characterized as the category of AI that will automatically be utilized with only minimum intervention from humans. Alternatively, a DL is a subcategory of ML that learns with massive data by implementing more neural network (NN) layers than traditional ML methods. Numerous evaluations under EEG-based BCI employ ML and signal processing [8]. A significant benefit of DL is that it will be performed individually in feature engineering. In this technique, the data are examined by exploring related features, followed by integrating those features to enable faster learning without a few categorical instructions. The motivation behind improving MI classification in BCIs stems from the requirement to enhance non-invasive techniques for translating brain activity into actionable outcomes [9]. Effectual MI classification can crucially advance assistive technologies, enabling more intuitive control of robotic arms, drones, and wheelchairs. By refining these methods, the study aims to give individuals with motor impairments greater autonomy and interaction with their environment. Employing advanced optimization and DL methods to improve classification accuracy can result in more reliable and effectual BCI models, fostering improved incorporation of technology with human cognitive processes and enhancing the quality of life for users [10].

This article presents a Boosted Harris Hawks Shuffled Shepherd Optimization Augmented Deep Learning (BHHSHO-DL) technique based on Motor Imagery Classification for BCI. The BHHSHO-DL technique mainly exploits the hyperparameter-tuned DL approach for MI identification for BCI. Initially, the BHHSHO-DL technique performs data preprocessing utilizing the wavelet packet decomposition (WPD) model. Besides, the enhanced densely connected networks (DenseNet) model extracts the complex and hierarchical feature patterns from the preprocessed data. Meanwhile, the BHHSHO technique-based hyperparameter tuning process is accomplished to elect optimal parameter values of the enhanced DenseNet model. Finally, the classification procedure is implemented by utilizing the convolutional autoencoder (CAE) model. The simulation value of the BHHSHO-DL methodology is performed on a benchmark dataset. The key contribution of the BHHSHO-DL approach is listed below.

- The WPD technique refines data preprocessing by decomposing complex signals into simpler, more manageable components. This methodology crucially improves the quality of the input data, making it more appropriate for analysis by subsequent models. By enhancing data clarity and structure, WPD supports more accurate and effectual modelling.
- The improved DenseNet methodology outperforms extracting complex and hierarchical features from preprocessed data. Employing its DL architecture, it captures intrinsic patterns with high precision. This advanced feature extraction enhances the overall performance and accuracy of the subsequent evaluation.
- Systematic tuning of hyperparameters in the improved DenseNet method optimizes parameter settings for enhanced performance. This meticulous alteration improves the technique’s accuracy and data, data analysis, and interpretation efficiency. The refined parameters result in more reliable and precise outcomes.
- Employing a CAE for hyperparameter tuning presents a novel technique for improving DenseNet model performance. The CAE technique’s capability to learn effective data representations allows for efficient parameter adjustment. This novel technique refines the methodology’s accuracy and effectiveness by utilizing the merits of the CAE model in representation learning.
- Incorporating a CAE for hyperparameter tuning presents a novel methodology that improves the performance of the DenseNet model. By implementing the CAE model’s capability to learn and represent data effectively, this technique refines the tuning process and crucially enhances the model’s accuracy. This innovative strategy departs from conventional hyperparameter tuning techniques, setting a new standard for optimization.

## 2. Literature review

In [11], an innovative deep transfer NN (DTNN) method was developed. Primarily, a filter bank has been employed. Afterwards, developed two domain adaptation components concurrently. The primary domain adaptation component chose the same source field information, and the 2^nd^ domain adaptation component decreased the variance among the target and source fields. In conclusion, two adversarial methods have been utilized to increase the precision and strength of the classification. In [12], a hybrid DL model for effectively exploring the EEG signal was developed. The method proficiently chose and utilized the convolutional NN (CNN) filters in the developed model to extract the significant multiple domain features. The redundant features could be mined using feature extraction to enhance the model’s effectiveness. In [13], an innovative modular and self-organized model was developed. A pattern recognition method was presented for transmitting the determined signals to make classes that signify considerations without prior preprocessing. A neuro-fuzzy component and a learning technique were used to analyze the model’s internal process. The whole learning method was dependent upon the ML technique. In [14], a multi-branch-CNN (MBCNN) method with a temporal convolution network (TCN), an end-wise DL method for decoding multiclass MI tasks was introduced. The technique initially employed the MBCNN method by employing diverse convolutional kernels. Next, the technique presented TCN for extracting more feature representations. The within-subject cross-session approach was implemented for validation.

Malibari et al. [15] developed an arithmetic optimizer with the RetinaNet-based DL method for MI classification (AORNDL-MIC) method under BCIs. The continuous wavelet transforms (CWT) and multiscale principal component analysis (MSPCA) techniques were employed. Besides, the DL-based RetinaNet could be utilized in the extraction process through the ID3 approach. Similarly, an arithmetical optimization algorithm (AOA) was applied to tune the hyperparameter of RetinaNet. In [16], an innovative technique employing artificial NN (ANN) architecture was proposed. Feature extraction methods are analyzed and compared. Four classification methods have been applied: LDA, KNN, and Quadratic Discriminant Analysis (QDA) developed ANN model. The study also included batch normalization levels in the presented ANN model to increase the learning accuracy and period of the NN. In [17], an effective CNN method for EEG-based MI classification was introduced. An automatic channel selection technique was developed to decrease the model difficulty, which relies on spatial filters and measures the activations and weights to 8-bit by slight precision loss. In [18], an EEG-Based temporal 1D-CNN (ETIODCNN) method was developed for categorizing MI. Primarily, the technique removed temporal relationships from EEG signals by presenting the core blocks. Next, the method employs the FC and global average pooling (GAP) layers to combine the temporal series features and achieve classification tasks. Sharma, Kim, and Gupta [19] compare conventional classification approaches with DL models, particularly Multi-Layered Perceptrons, for EEG MI tasks. It exhibits that SVM technique gives the quickest training and prediction speeds while maintaining comparable accuracy to conventional techniques.

In [20], the authors introduce a multiscale CNN (MS-CNN) technique that extracts key features from diverse EEG frequency bands for MI BCI classification. The model improves accuracy by incorporating user-specific features and data augmentation methods to enhance robustness. Roy [21] proposes a transfer learning (TL)-based multiscale feature fused CNN (MSFFCNN) technique for multiclass MI classification, capturing features from diverse EEG frequency bands. Kumari et al. [22] present a hybrid optimization technique incorporating War Strategy Optimization (WSO) and Chimp Optimization Algorithm (ChOA) models to improve classification performance. The two-tier DL method, comprising a CNN for temporal features and a modified Deep NN (M-DNN) technique for spatial characteristics, improves BCI control through optimal channel selection and advanced optimization. Xie and Oniga [23] propose an integrated time-frequency domain data enhancement methodology. The approach also presents a parallel CNN that processes raw EEG images and those transformed via the CWT technique. Echtioui et al. [24] introduce an ANN model to improve the classification performance of MI. Feature extraction methods, comprising time domain parameters and WPD, are also associated. Alsuradi et al. [25] present Shapley-informed augmentation to enhance within-subject accuracy, depending on data-driven evaluation that detected inconsistent temporal features across sessions for finger MI. Arı and Taçgın [26] developed the No-Filter EEG (NF-EEG) method, a robust CNN that classifies multiclass MI signals directly from raw data without preprocessing. The technique also employed input reshaping and utilized diverse data augmentation models.

The existing studies on MI classification techniques encounter various limitations. Models employing dual domain adaptation and adversarial approaches may need more computational complexity and scalability. Hybrid DL techniques utilizing convolutional filters may face problems with redundant features, affecting their efficiency. Models lacking preprocessing, namely modular and self-organized methods, may experience mitigated accuracy and robustness. Multi-branch CNNs with TCNs can be computationally intensive, while methodologies incorporating arithmetic optimization and DL mostly encounter threats with hyperparameter tuning. ANN methods with feature extraction may need to address overfitting or generalization adequately. Automatic channel selection in the CNN technique might result in precision loss, and temporal 1D-CNN techniques may need to be more accurate in their more accurate temporal relationships. Methods encompassing multiscale CNNs or TL may need help with dataset variability and generalization, and hybrid optimization approaches can have difficulties balancing exploration and exploitation. Time-frequency domain enhancement models may also encounter efficiency problems, and techniques, namely No-Filter EEG, may be limited by the need for more preprocessing, affecting their capability to handle noisy data effectively. Existing MI classification techniques encounter threats: computational complexity, limited generalization across various datasets, and insufficient handling of noisy or inconsistent data. Moreover, there is a requirement for more efficient feature extraction and data augmentation methods that can enhance accuracy and robustness while mitigating dependence on extensive preprocessing.

## 3. The proposed method

**Fig 1:**
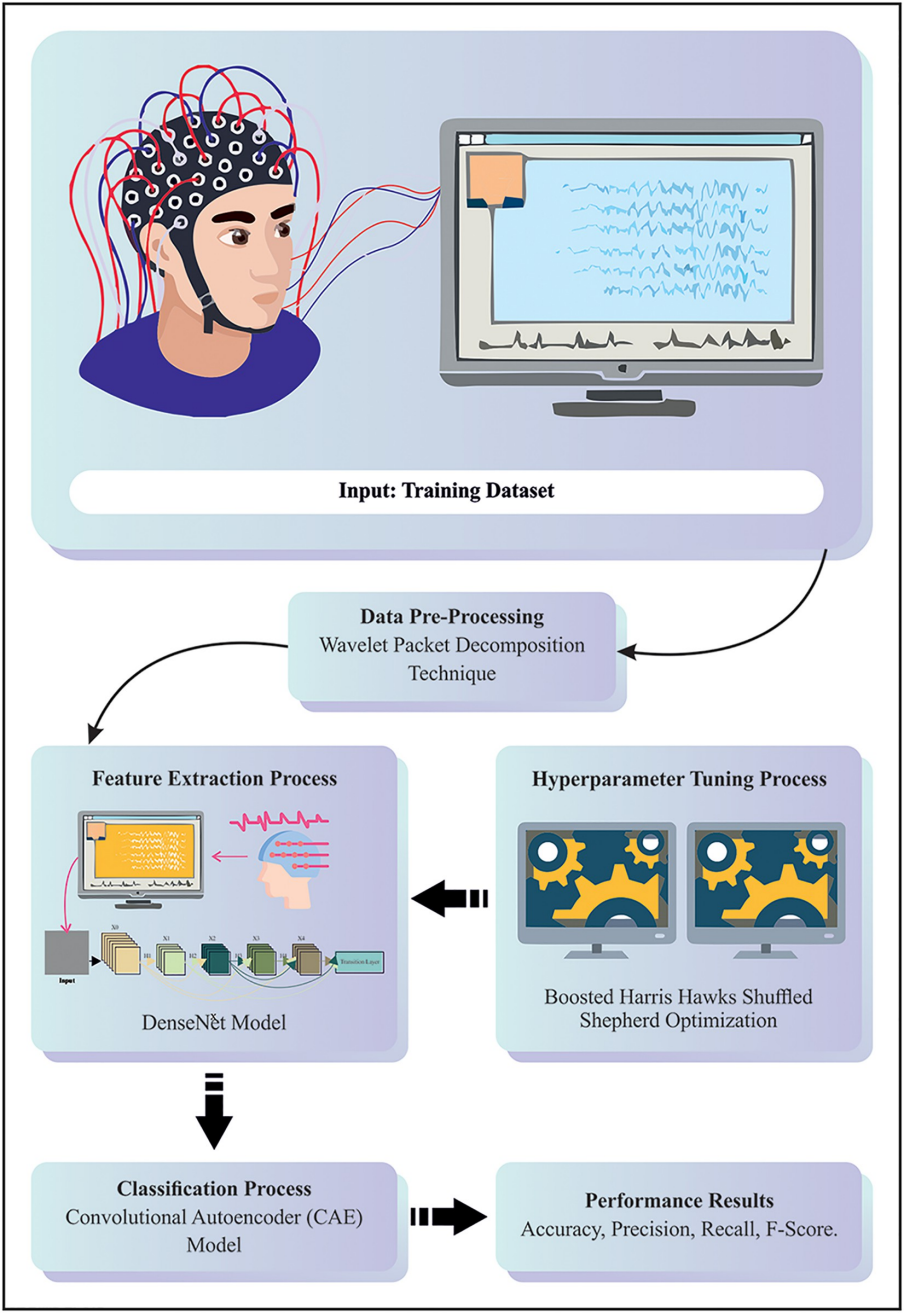
Overall flow of BHHSHO-DL technique.

This study presents a unique BHHSHO-DL technique-based MI classification for BCI. The BHHSHO-DL technique mainly exploits a hyperparameter-tuned DL model for MI identification for BCI. To obtain this, the BHHSHO-DL method followed four main methods: WPD-based preprocessing, DenseNet-based feature extractor, BHHSHO-based hyper-parameter tuning, and CAE-based classification. Fig 1 illustrates the complete flow from the BHHSHO-DL model.

### 3.1. Data preprocessing

Initially, the BHHSHO-DL technique performs data preprocessing using the WPD technique. The WPD model is a developed model dependent upon wavelet decomposition [27]. The WPD model is highly advantageous for preprocessing because it can effectively handle non-stationary signals. Unlike conventional Fourier-based techniques, WPD decomposes data into diverse frequency sub-bands with both time and frequency resolution, making it adept at capturing transient and localized features. This elaborated decomposition allows for precise noise reduction and artefact removal, specifically in complex signals like EEG, where diverse kinds of noise affect several frequency ranges. Furthermore, WPD’s multi-resolution analysis eases the extraction of relevant features while conserving significant signal characteristics. Related to other models, WPD provides a more refined and adaptable preprocessing technique, improving the data’s quality and accuracy for subsequent evaluation. Preprocessing with the WPD method effectually addresses noise and artefacts in EEG data by decomposing the signal into various frequency sub-bands.

**Fig 2:**
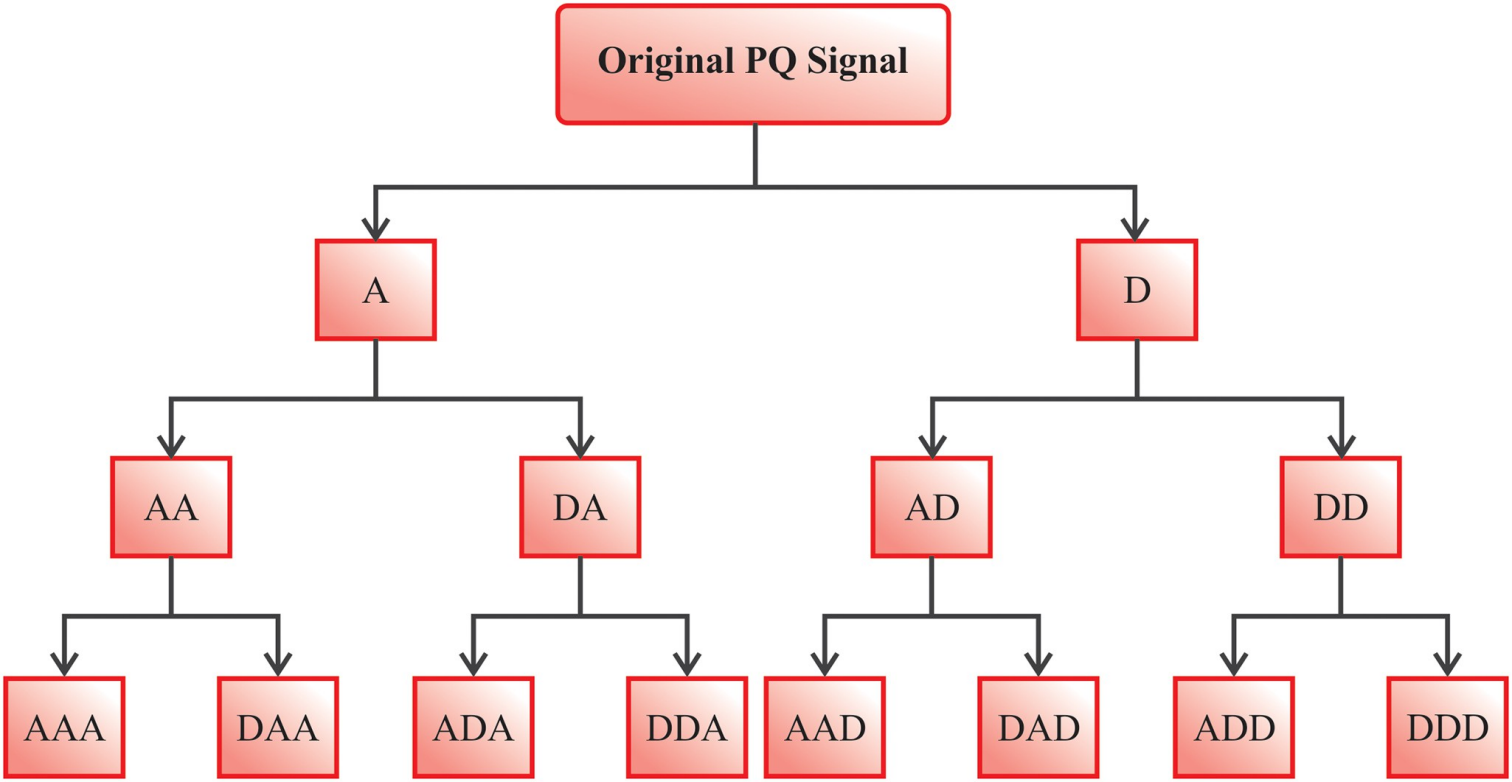
Workflow of WPD approach.

This decomposition isolates and removes noise and artefacts from specific frequency ranges, enhancing the signal-to-noise ratio. By concentrating on the most relevant frequency components and reconstructing the signal from these clean sub-bands, WPD improves data quality while conserving critical data for subsequent analysis. This methodology confirms that the cleaned EEG data is more accurate and reliable for additional processing and interpretation. The capability of the WPD to manage non-stationary signals is crucial for EEG processing, as EEG data often exhibit rapid fluctuations and transient events that need precise evaluation. This capability confirms that significant features are captured while minimizing the impact of noise and artefacts. Moreover, multi-resolution analysis of the WPD model facilitates the superior decomposition of EEG signals compared to Independent Component Analysis (ICA), which may encounter difficulty ineffectually isolating transient noise, ultimately improving the clarity and reliability of the data. Fig 2 portrays the overall structure of the WPD model.

This model includes the low-resolution of WPD maximum frequency. It examines the signals in a better way. WPD physically selects the proposer frequency range equivalent to the signal band for dissimilar signals, enhancing time-frequency resolution. WPD is a superior efficacy from the limited analysis. It eliminates the annoying data and upholds the feature database, i.e., it is beneficial to identify and better state the EEG signal database. Many resolution studies decay the *L*(*R*) of Hilbert space as to the orthogonal sum of wavelet sub-space *W*_*l*_ dependent upon the *l* scaling factor. A novel sub-space $U_{l}^{m}$ signifies the *V*_*l*_ scale space and *W*_*l*_ wavelet subspace.


$$t_{U_{1}^{1}=W_{l},l\in Z}^{U_{l}^{0}=V_{l},l\in Z}$$ (1)

The orthogonal decomposition of Hilbert space *V*_1_ ⊕ *W*_1_ is expressed by:

$$U_{l}+1^{0}=U_{l}^{0}⊕U_{l},\;l\in Z.$$ (2)

Define the sub-space $U_{l}^{m}$ as an end function space *u*_*m*_(*t*), such that *u*(*t*) achieves:

$$\left\{\begin{array}{l} u_{2m}\left(t\right)=\sqrt{2}\;\sum_{k\epsilon Z}\;h\left(k\right)u_{2m}\left(2t-k\right)\\ u_{2m+1}\left(t\right)=\sqrt{2}\;\sum_{k\epsilon Z}\;g\left(k\right)u_{2m}\left(2t-k\right)\;’ \end{array}\right.$$ (3)

In Eq (3), (*k*) = (−1)^*k*^*h*(1 − *k*), *g*(*k*), and *h*(*k*) represent the co-efficient of lower and higher pass filtering, which is orthogonal to another.

If *m* = 0, from Eq (3), the following results are obtained:

$$t_{u_{1}\left(t\right)=\sum_{k\epsilon Z}\;g\left(k\right)u_{0}\left(2t-k\right)}^{u_{0}\left(t\right)=\sum_{k\epsilon Z}\;h\left(k\right)u_{0}\left(2t-k\right)}.$$ (4)

During many resolution analysis processes, the function of wavelet basis *ϕ*(*t*) and scale *ψ*(*t*) achieve:

$$\left\{\begin{array}{c} \varphi \left(t\right)=\sum_{k\epsilon Z}\;g\left(k\right)\psi \left(2t-k\right)\\ \psi \left(t\right)=\sum_{k\epsilon Z}\;h\left(k\right)\psi \left(2t-k\right) \end{array}\right.$$ (5)

From the formulation, given that *ϕ*(*t*) = *u*_1_(*t*) and (*t*) = *u*_0_(*t*). Thus {*u*_*m*_(*t*)}_*m*∈*Z*_ signify orthogonal wavelet packets. The expression of WPD co-efficient has been determined below:

$$\left\{\begin{array}{l} d_{i}^{l+1,2m}=\sum_{k\epsilon Z}\;h\left(k-2i\right)\;d_{k}^{l,m}\\ d_{i}^{l+1,2m+1}=\sum_{k\epsilon Z}\;g\left(k-2i\right)\;d_{k}^{l,m} \end{array}\right.$$ (6)

The WPD is a superior resolution of time-frequency in lower and higher-frequency. The EEG signals data is assumed to be the wavelet packet co-efficient on each decomposition scaling, which is various and considered a removing feature. The EEG is decayed by employing wavelet packets to attain wavelet coefficients on dissimilar measures. The re-formed EEG signals of $S_{4}^{0},S_{4}^{1},S_{4}^{2}$, and $S_{4}^{3}$ are used as input for the classification of EEG.

### 3.2. DenseNet-based feature extraction

**Fig 3:**
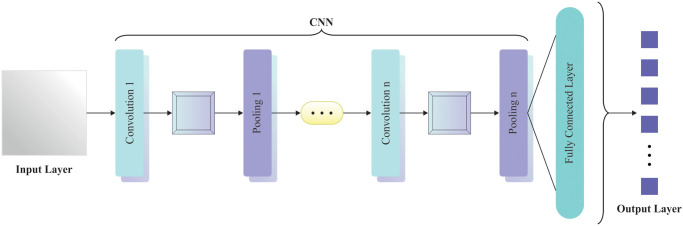
Framework of DenseNet model.

At this stage, the enhanced DenseNet model extracts the complex and hierarchical feature patterns from the preprocessed data [28]. The DenseNet-based feature extraction model is highly effective due to its unique architecture, which promotes efficient feature reuse and deep network training. DenseNet connects each layer to every other layer feed-forward, allowing for better gradient flow and reducing the vanishing gradient issue. This dense connectivity eases the extraction of complex and hierarchical features, enhancing the capability of the technique to capture intrinsic patterns in the data. Moreover, DenseNet’s compact architecture mitigates the number of parameters, which reduces computational cost and overfitting. Compared to other methods, DenseNet’s effectual feature propagation and reuse contribute to more accurate and robust feature extraction, particularly in complex and high-dimensional datasets. Fig 3 demonstrates the framework of DenseNet.

DenseNet comprises a classification layer, three transition layers, four dense blocks, *a* 3x3 max pooling layer, and a 7x7 convolution layer with a stride of 2. Each dense block carries out 3x3 and 1x1 convolutions and follows the transition layer. The transition layer comprises a 2x2 average pooling layer and 1*x*1 convolutions. The last classification layer contains fully connected layers and a 7*x*7 GAP layer that uses an activation function like softmax. DenseNet is the establishment of dense connections ensuring the high data flow among layers. Unlike traditional CNN with L and L connections, DenseNet contains $\frac{L\left(L+1\right)}{2}$ direct association with *L* layers. Assume the input image is *x*_0_, the CNN comprises five layers, *x*_*i*_(*I* = 0, 1, 2, ⋯ 5) denotes the mapping feature of the *ith l*ayer, *H*_*i*_() indicates the nonlinear conversion, which may involve pooling, convolution, batch normalization, activation function, etc. Hence, the outcome of the 5^th^ layer is a nonlinear conversion of resultant mapping features.


$$x_{5}=H_{5}\left(\left[x_{0},\;x_{1},\cdots x_{4}\right]\right).$$ (7)

The attention module is a resource allocation method and is classified into pixel attention, multi-stage attention, channel attention, etc. This study used a squeeze and excitation (SE) block to learn the feature weights depending on the loss, such that the efficient feature maps have greater weight. The excitation function acquires the weighted to explicitly model the connection among feature channels and produces weight for all the feature channels via parameter. For any given conversion, *F*_*tr*_ mapping the input *X*(*X* ∈ *R*^*H*′×*W*′×*C*′^) into the feature maps *U* where *U* ∈ *R*^*H*×*W*×*C*^, a respective SE module is generated to perform feature recalibration. Firstly, the feature *U* is passed through the squeeze function *F*_*sq*_ that compresses *U* into 1 × 1 × *C* features. Then, the features from *F*_*sq*_ were excited using the excitation function *F*_*ex*_. Lastly, the recalibration feature $\tilde{X}\left(\tilde{X}\in R^{H^{\prime }\times W^{\prime }\times C^{\prime }}\right)$ is obtained through *F*_*scale*_, where *F*_*Scale*_ implies that the weighted of excitation output is sequentially weight to the prior feature channel by multiplication and concludes the recalibration of novel features in the channel size.

### 3.3. Hyperparameter tuning process

In this work, the BHHSHO approach-based parameter tuning process occurs to elect optimal parameter values of the enhanced DenseNet model [29]. The BHHSHO method is appropriate for hyperparameter tuning due to its robust search capabilities and improved convergence properties. By incorporating the HHO with shuffled shepherd strategies, BHHSHO efficiently balances exploration and exploitation during the tuning process. This hybrid technique enhances the capability to escape local optima and attain a more global search, resulting in optimal parameter settings. The improved mechanism of the model additionally refines this process, accelerating convergence and enhancing the likelihood of finding greater hyperparameter values. Related to conventional models, the BHHSHO technique presents a more adaptive and effectual solution, making it highly efficient for intrinsic and high-dimensional optimization issues.

**Fig 4:**
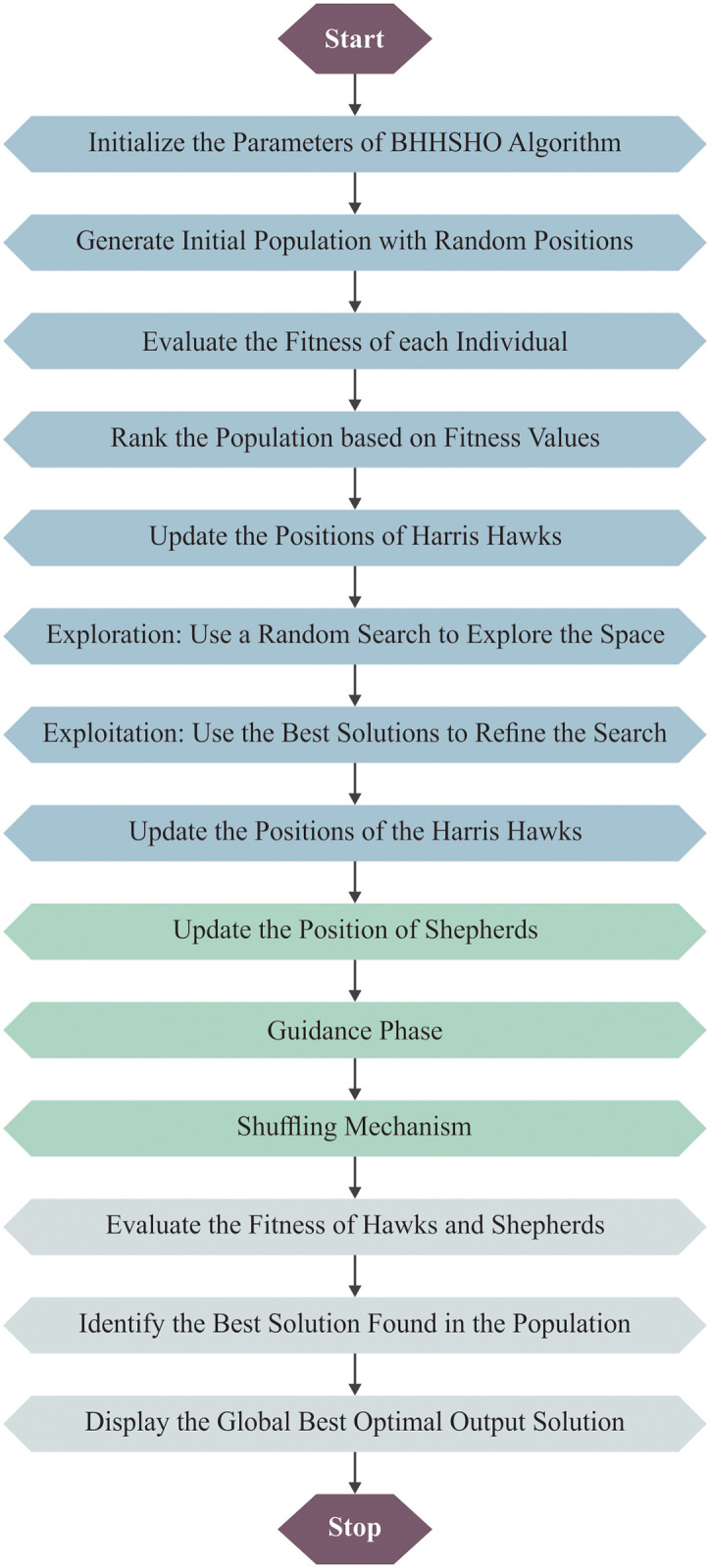
Steps involved in the BHHSHO technique.

Hyperparameters such as learning rate and batch size play a significant role in the performance of the BHHSHO model. The learning rate determines how quickly the model adjusts to the error during training; an optimal rate facilitates faster convergence and reduces loss effectually. Meanwhile, batch size influences the stability of gradient updates and affects the capability of the technique to generalize. In this study, the learning rate emerged as the most impactful hyperparameter, substantially improving the method’s accuracy and reducing loss compared to other settings, accentuating the significance of careful hyperparameter tuning in attaining optimal performance metrics. Fig 4 illustrates the steps involved in the BHHSHO model.

BHHSSO is a fusion of HHO and the SSOA. HHO’s primary objective is to mimic hawks’ normal hunting behaviour and prey movement to detect solutions for single-objective problems. According to the BHHSSO approach, the SSOA implemented the upgrade solution for HHO.

#### Exploration stage

This phase describes the location of hawks’ when finding the prey. It relies on two strategies. The former explains how hawks find their prey according to the actual position (*S*_*i*_, *I* = 1, 2, …, *N*), *N* denotes the hawks’ number, which is depicted in Eq (8). The latter explains how hawks find their prey according to the placement on a random tree (*S*_*rand*_) given in Eq (9), where *S*_*i*_(*t* + 1) shows the location update of hawks in subsequent iteration *t*, *S*_*rand*_(*t*) specifies the present position of Hawks, *r*_1_, *r*_2_, *r*_3_, *r*_4_ and *q* is the random number that lies between zero and one. In Eq (10), *S*_*m*_(*t*) specifies the mean position for the entire hawks. However, according to the BHHSSO method, the (*S*_*rand*_) computation relies on the best, worst, and existing location, as shown in Eq (10). *S*_*prey*_(*t*) denotes the prey location.


$$S_{i}\left(t+1\right)=\left\{\begin{array}{ll} S_{rand}\left(t\right)-r_{1}\left|S_{rand}\left(t\right)-2r_{2}S\left(t\right)\right| & q\geq 0.5\\ \left(S_{prey}\left(t\right)-S_{m}\left(t\right)-U\right), & q≺0.5 \end{array}\right.$$ (8)

Here, *U* = *r*_3_(*lb* + *r*_4_(*ub* − *lb*))

$$S\left(t\right)=\frac{\Sigma_{i=1}^{N}S_{i}\left(t\right)}{N}$$ (9)

*S*_*rand*_(*t*) is evaluated in Eq (10) according to the BHHSSO.


$$S_{rand}\left(t\right)=\frac{S_{best}\left(t\right)+S_{worst}\left(t\right)+S}{3}$$ (10)

#### Shift from exploration to exploitation

This stage of HHO attempts to model and define how hawks change their behaviour from the exploration to exploitation stages. Such behaviours are reliant on the escaping energy of prey (*Eg*), as shown below:

$$Eg=2Eg_{0}\left(1-\frac{t}{t^{*}}\right)$$ (11)

(*Eg*) is computed in Eq (12) according to BHHSSO, whereas *C* indicates the chaotic randomly produced integer evaluated by the logistic map function. A polynomial map of degree 2, termed the logistic map in Eq (13), was often utilized as a typical example of how a nonlinear dynamical equation might produce complex, chaotic behaviour.


$$Eg=2CEg_{0}\left(1-\frac{t}{t^{*}}\right)$$ (12)

$$\Phi_{k+1}=a\Phi_{k}\left(1-\Phi_{k}\right)$$ (13)

*Eg* indicates the escape energy; *Eg*_0_ symbolizes the initial energy of prey; *t* shows the iteration count; and t* signifies the maximal iteration.

#### Exploitation stage

The prey escape behaviour and hawk hunting strategy are two primary components of this stage. In the exploitation stage, four strategies were followed:

- Soft besiege
- Hard besiege
- Soft besiege with progressive quick dives
- Hard besiege with progressive quick dives.

#### Soft besiege

A gentle besiege occurs when |*Eg*| ≥ 0.5 and *r* ≥ 0.5. The modelling of these behaviours is depicted in Eq (19), whereas *ΔS*(*t*) indicates the differences between the new location at *t* iteration and the location vector of the rabbit. The prey escaping strategy, *Jp* = 2(1 − *r*_5_), randomly varies at all the iterations. *r*_5_ shows the random number in [0, 1].


$$S_{i}\left(t+1\right)=\Delta S\left(t\right)-Eg\left|IpS_{prey}\left(t\right)-S\left(t\right)\right|$$ (14)

$$\Delta S\left(t\right)=S_{prey}\left(t\right)-S\left(t\right)$$ (15)

#### Hard besiege

If |*Eg*| ≺ 0.5 and *r* ≥ 0.5, this technique leads to 2 hard besieges. The location updating of hawks is given in Eq (16).


$$S_{i}\left(t+1\right)=S_{prey}\left(t\right)-Eg\left|\Delta S\left(t\right)\right|$$ (16)

The SSOA performs the position update of hawks according to BHHSSO.


$$S_{i}^{new}=S_{i}^{best}+stepsize_{i}+Levy\left(\beta \right)$$ (17)

#### Soft besiege with progressive quick dives

If |*Eg*| ≥ 0.5, the prey has enough energy for escaping, and *r* ≺ 0.5, then hawks are still constructing a soft besiege. This technique upgrades a hawk’s location. Team quick dives based on the levy’s flight are performed to increase the exploitation ability. *R* specifies the problem dimension, *LF* shows the levy’s flight function, *Q* implies the arbitrary vector with 1×*R* size, and *V* specifies the exploitation ability.


$$U=S_{prey}\left(t\right)-Eg\left|IpS_{prey}\left(t\right)-S\left(t\right)\right|$$ (18)

$$V=U+Q\times LF\left(R\right)$$ (19)

$$LF\left(S\right)=\frac{l\times \sigma }{\left|m\right|\frac{1}{\gamma }},\sigma ={\left(\frac{\Gamma \left(1+\gamma \right)\times \text{sin}\;\left(\frac{\pi \gamma }{2}\right)}{\Gamma \left(\frac{1+\gamma }{2}\right)\times \gamma \times 2^{\left(\frac{\gamma -1}{2}\right)}}\right)}^{\frac{1}{\gamma }}$$ (20)

Where *l* and *m* are the random numbers between zero and one, *γ* specifies the constant number set as 1.5. Eq (21) determines the location update of hawks in soft besiege with progressive quick dives.


$$S\left(t+1\right)=\left\{\begin{array}{l} U\;if\;Z\left(U\right)≺Z\left(S\left(t\right)\right)\\ V\;if\;Z\left(V\right)≺Z\left(S\left(t\right)\right) \end{array}\right.$$ (21)

#### Hard besiege having progressive quick dives

If *r* ≺ 0.5, the hawks constructed a hard besiege; if |*Eg*| ≺ 0.5, prey cannot escape. This strategy relies on the hard besiege provided in Eq (22).


$$S\left(t+1\right)=\left\{\begin{array}{l} U\prime \;if\;Z\left(U\prime \right)≺Z\left(S\left(t\right)\right)\\ V\prime \;if\;Z\left(V\prime \right)≺Z\left(S\left(t\right)\right) \end{array}\right.$$ (22)

Fitness choice is a significant factor in managing the efficiency of the BHHSHO approach. The choice of hyperparameter contains the solution of the encoded system to measure the effectiveness of candidate outcomes. During this case, the BHHSHO methodology assumes accuracy as the primary condition to plan the FF, which is defined as:

$$Fitness=\text{max}\;\left(P\right)$$ (23)

$$P=\frac{TP}{TP+FP}$$ (24)

*FP* and *TP* define false and true positive values.

#### Convergence curve for BHHSHO optimization

**Fig 5:**
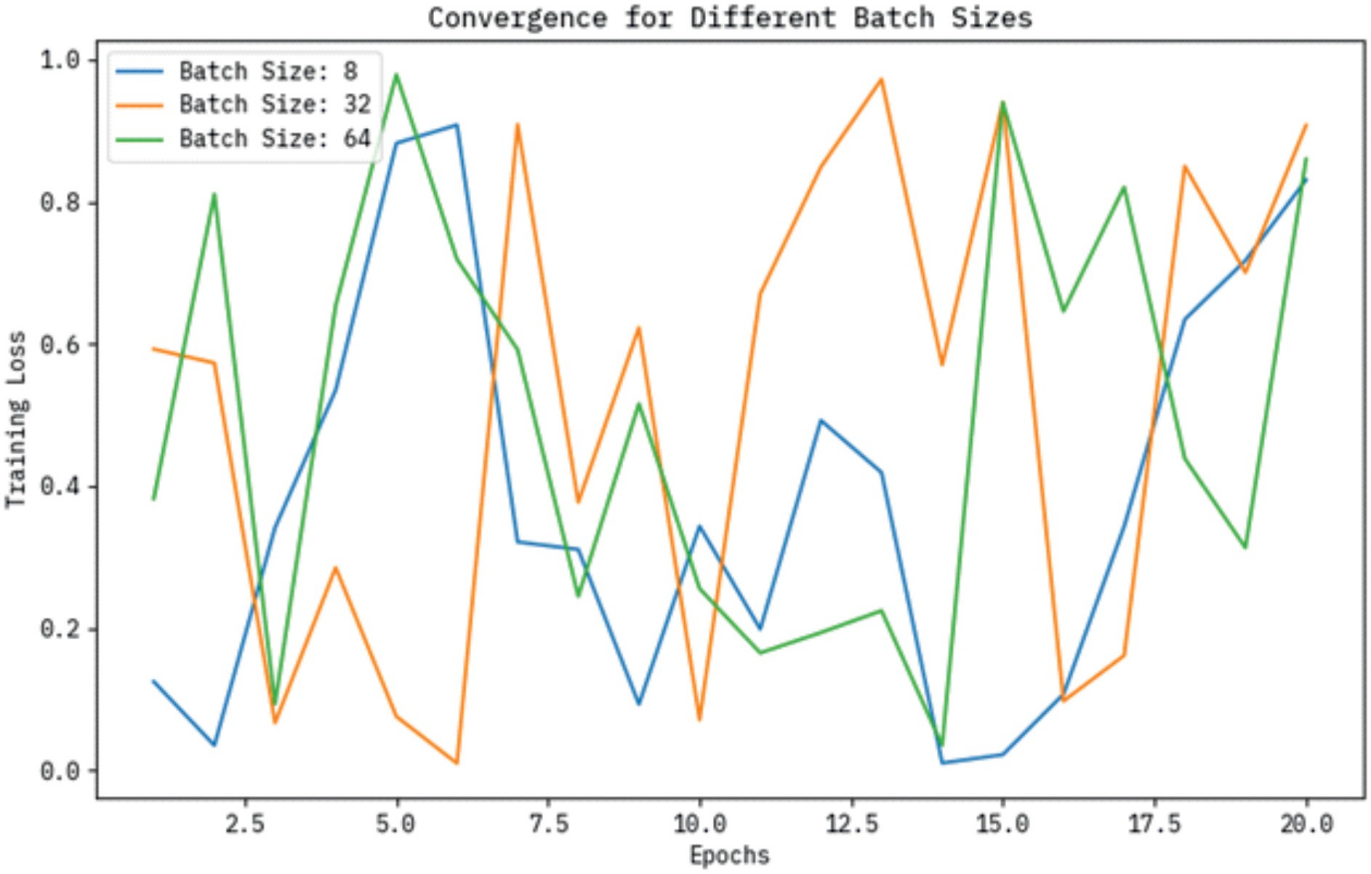
Convergence curve of BHHSHO model for different batch sizes.

The convergence curves for the BHHSHO-DL technique are represented, demonstrating the effects of varying batch sizes, learning rates, and the number of layers. These curves emphasize how each parameter influences the model’s convergence behaviour, accentuating differences in speed and stability. The optimal configurations that result in efficient training and enhanced performance can be detected by analyzing these discrepancies. The training loss convergence curves for diverse batch sizes such as 8, 32, and 64 are depicted over a range of epochs in Fig 5. As illustrated, the loss decreases more rapidly with batch sizes of 32 and 64 compared to a batch size of 8, suggesting that larger batch sizes facilitate more stable training and faster convergence. This trend underscores the impact of batch size on the model’s learning efficiency throughout the training process.

**Fig 6:**
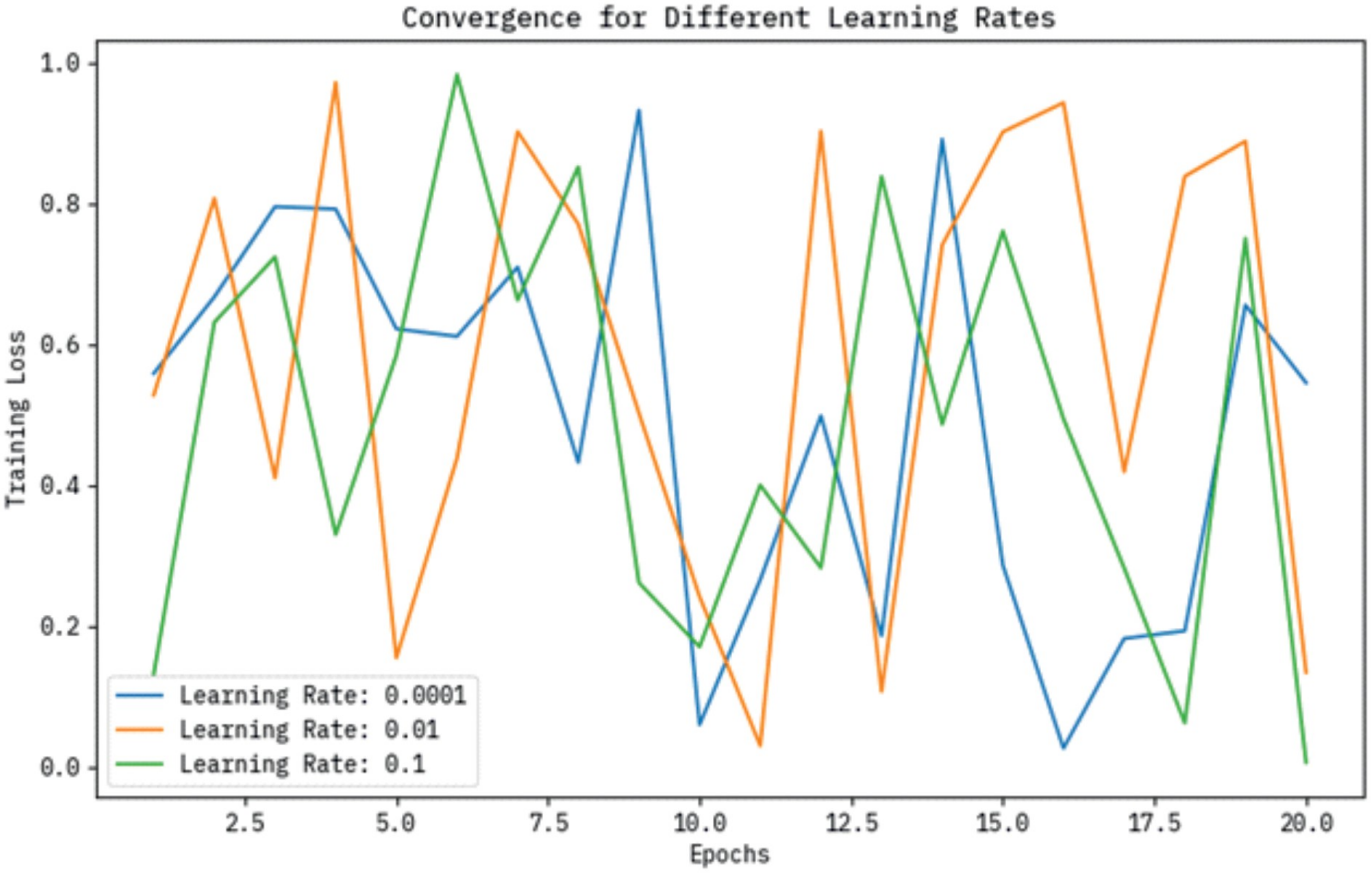
Convergence curve of the BHHSHO model for different learning rates.

The training loss convergence curves for varying learning rates, namely 0.0001, 0.01, and 0.1, are presented over a series of epochs in Fig 6. The graph shows that a learning rate of 0.01 results in the most efficient loss reduction, while the lowest learning rate (0.0001) exhibits slower convergence. On the contrary, a learning rate of 0.1 depicts initial instability, representing that finding an optimal learning rate is significant for attaining effectual and stable training performance.

**Fig 7:**
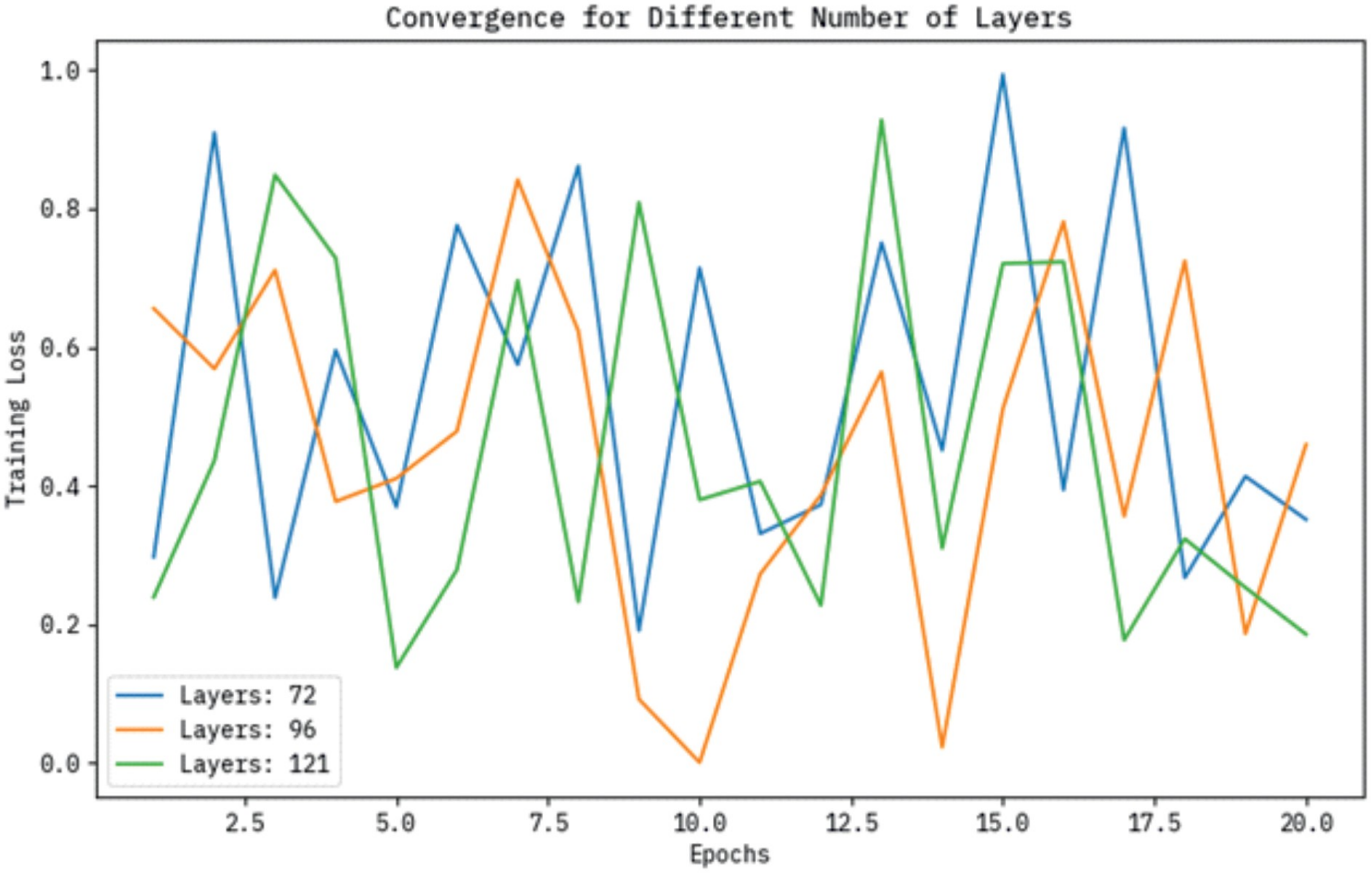
Convergence curve of BHHSHO model for different number of layers.

The training loss convergence curves for models with different numbers of layers, namely 72, 96, and 121, are portrayed throughout several epochs in Fig 7. The graph depicts that the technique with 96 layers attains the most balanced and effectual reduction in loss, while the model with 121 layers experiences diminishing returns and slower convergence. On the contrary, the 72-layer model illustrates a relatively quick decrease in loss but may lack the depth required for capturing intrinsic patterns. This emphasizes optimizing the number of layers to balance performance and training effectualness.

### 3.4. CAE-based classification process

Eventually, the classification procedure is implemented by utilizing the CAE model [30]. The CAE method is advantageous for classification tasks because it can learn effectual, hierarchical feature representations from raw data. By encoding input data into a compressed latent space and decoding it, CAEs capture crucial patterns and reduce dimensionality, improving the quality of features given in classification models. This methodology enhances feature extraction and reduces noise and redundancy, providing more precise and robust classification outcomes. Furthermore, CAEs are effectual in handling complex, high-dimensional data, making them superior to conventional techniques that might face difficulty with such threats. Their unsupervised and representation learning capacity gives them a crucial edge in attaining improved performance and generalization in classification tasks.

**Fig 8:**
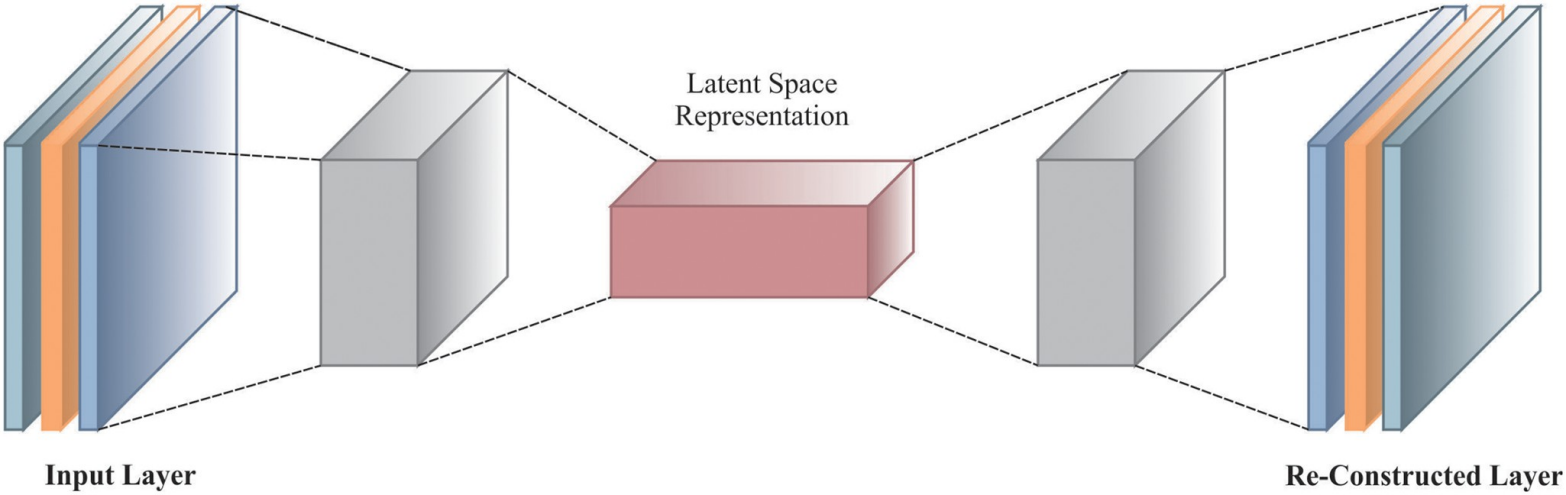
Structure of CAE technique.

This model also outperforms dimensionality reduction and hierarchical feature learning, making it specifically effectual for EEG-based motor imagery (MI) classification. By capturing spatial and temporal patterns within the data, CAEs can automatically learn relevant features at various levels of abstraction, improving the accuracy of the classification. On the contrary, standard classifiers such as SVM depend heavily on predefined features, which may only partially capture the intrinsic relationships in EEG signals, resulting in suboptimal performance. This adaptive learning capability of CAEs allows for more robust and complex representations of the EEG data. Fig 8 specifies the structure of the CAE method.

CAE combines the local convolution connection and the AE, adding a reconstructed convolutional input. The output value is reconstructed via the inverse convolution process called the convolution encoder. Next, the conversion process of convolution from the input mapping feature to the output is known as the convolution decoder. Furthermore, the parameters of encoding and decoding operations are computed through the typical AE unsupervised greedy training. The CAE operation lay, where *f*′(∙) and *f*(∙) are the convolutional decoder and encoder operations. Input feature map *x* ∈ *R*^*n*×l×l^ is attained from the input layer or prior layer. The dimension of the feature map is *l* × *l* pixels, and it encompasses *n* feature maps. The CAE operation consists of *m* convolution kernels and the resultant layer output *m* mapping feature. *n* denotes the number of input channels once the input mapping feature is generated in the input layer. *n* indicates the number of outcome mapping features from the prior layer when the input mapping features are from the prior layer. The convolution kernel size is *d*×*d*, whereas *b* ∈ *R*^*m*^ and *W* = {*w*_*j*_, *j* = 1, 2, ⋯, *m*} characterize the parameters of the convolutional encoder, *d* ≤ *l*. $\theta =\left\{W,\hat{W},\;b,\hat{b}\right\}$ signifies the parameter of CAE layer to be learned, *w*_*j*_ ∈ *R*^*n*×*l*×*l*^ is represented as a vector $w_{j}\in R^{nl^{2}}$. And $\hat{W}=\left\{{\hat{w}}_{j},\;j=1,2,\;\cdots ,\;m\right\}$ and $\hat{b}$ are the parameters of the convolution decoder, where $\hat{b}\in R^{nl^{2}}$, ${\hat{w}}_{j}\in R^{1\times nl^{2}}$. Firstly, the input images are encoded so that every time a *d* × *d* pixel patches *x*_*i*_, *I* = 1, 2, ⋯, *p*, is chosen from the input images, and later, the weighted *w*_*j*_ of the *j*^*th*^ convolutional kernel can be applied for convolution computation. Lastly, the neuron value *o*_*ij*_, *j* = 1, 2, ⋯, *m* is computed from the output layer.


$$o_{ij}=f\left(x_{i}\right)=\sigma \left(w_{j}\cdot x_{i}+b\right)$$ (25)

Where *σ* is a nonlinear activation function,

$$Relu\;\left(x\right)=\left\{\begin{array}{l} x\;x\geq 0\\ 0\;x<0 \end{array}\right.$$ (26)

Then, the *o*_*ij*_ output from the convolution decoder is encoded, and the *x*_*i*_ is recreated through the *o*_*ij*_ for the ${\hat{x}}_{i}$.


$${\hat{x}}_{i}=f\prime \left(o_{ij}\right)=\phi \left({\hat{w}}_{i}\cdot o_{ij}+\hat{b}\right)$$ (27)

After each convolutional encoder and decoder, ${\hat{x}}_{i}$ is generated. *P* patch is attained from the reconstructed function with *d*×*d*. The MSE is used among the reconstructed patch of image ${\hat{x}}_{i}$ (*i* = 1, 2, ⋯ *p*) and the original input image *x*_*i*_ (*i* = 1, 2, ⋯ *p*) as the cost function. Moreover, the reconstruction error is defined in Eq (29), and the cost function is defined in Eq (28).


$$J_{CAE}\left(\theta \right)=\frac{1}{p}\sum_{i=1}^{p}L\left[x_{i},{\hat{x}}_{i}\right]$$ (28)

$$L_{CAE}\left[x_{i},{\hat{x}}_{i}\right]=∥x_{i}-{\hat{x}}_{i}{∥}^{2}=∥x_{i}-\phi \left(\sigma \left(x_{i}\right)\right){∥}^{2}$$ (29)

**Fig 9:**
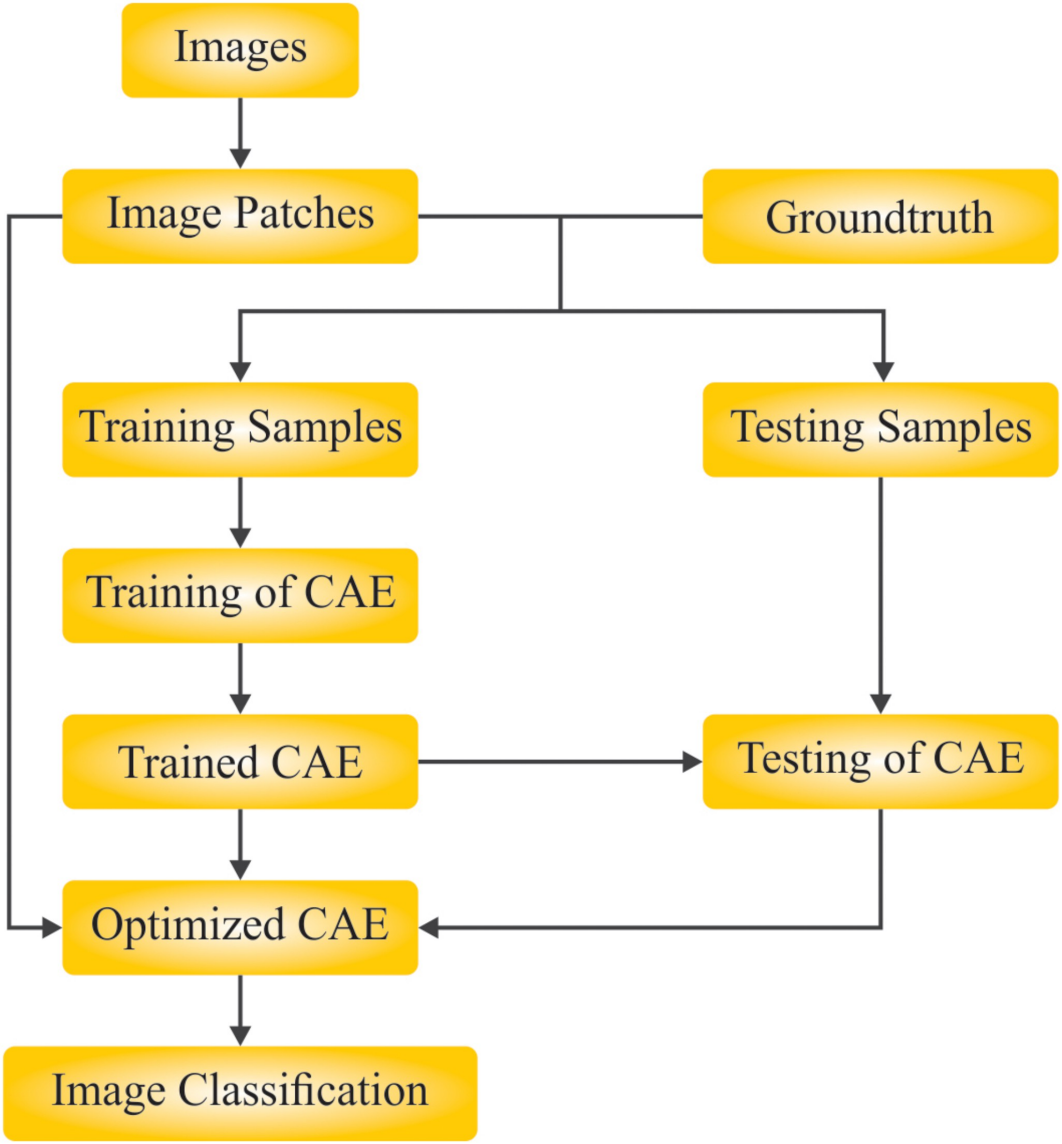
Flowchart of CAE technique.

Stochastic gradient descent (SGD) can be deployed to reduce the weights and errors, enhancing the CAE layer. Lastly, the trained parameter outputs the feature map, which is transferred to the following layer. Fig 9 illustrates the flowchart of the CAE model.

## 4. Performance validation

This section investigates the performance of the BHHSHO-DL methodology on the BCI Competition (BCIC)-III database [31] and BCIC-IV database. The chosen databases are preferred for MI classification due to their high-quality, diverse EEG recordings and well-defined experimental protocols. These datasets present extensive and varied MI tasks, making them ideal for developing and benchmarking robust classification models. Their established utilization in the BCI community confirms reliability and comparability with existing research.

**Fig 10:**
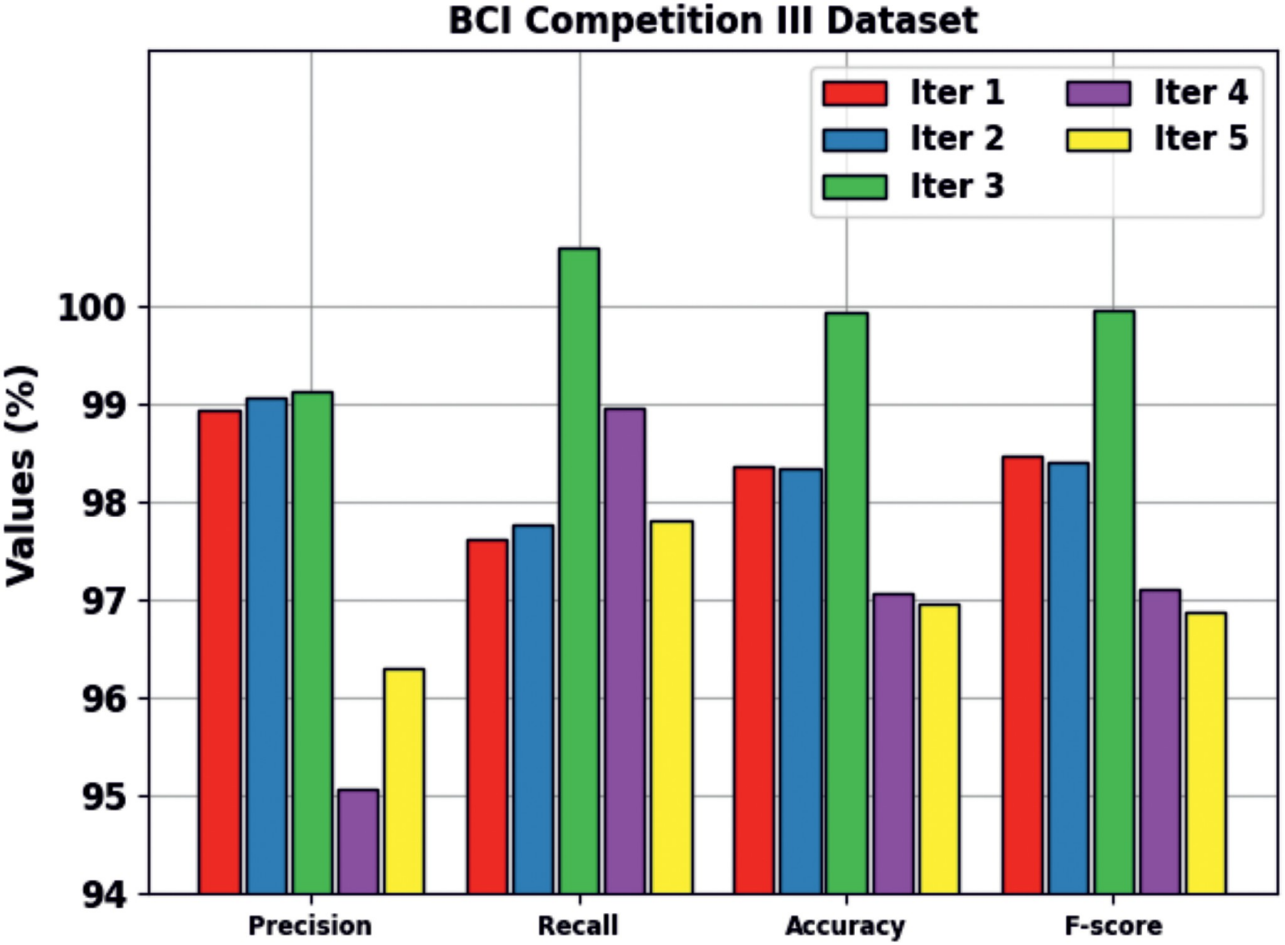
Classification outcome of the BHHSHO-DL model with BCIC-III database.

Table 1 and Fig 10 portray the classification outcomes offered by the BHHSHO-DL technique on the applied BCIC-III database. These accomplished values denote that the BHHSHO-DL method offers enhanced performance under all iterations. It is noticed that the BHHSHO-DL method obtains an average *prec*_*n*_ of 97.70%, *reca*_*l*_ of 98.56%, *accu*_*y*_ of 98.15%, and *F*_*score*_ of 98.17%.

**Table 1:** Classification results of the BHHSHO-DL model under the BCIC-III database.

BCIC-III Database				
No. of iterations	*Prec*_*n*_	*Reca*_*l*_	*Accu*_*y*_	*F*_*score*_
Iter 1	98.95	97.63	98.37	98.48
Iter 2	99.07	97.78	98.36	98.42
Iter 3	99.13	100.61	99.95	99.96
Iter 4	95.06	98.96	97.07	97.12
Iter 5	96.30	97.82	96.97	96.88
**Average**	**97.70**	**98.56**	**98.15**	**98.17**

**Fig 11:**
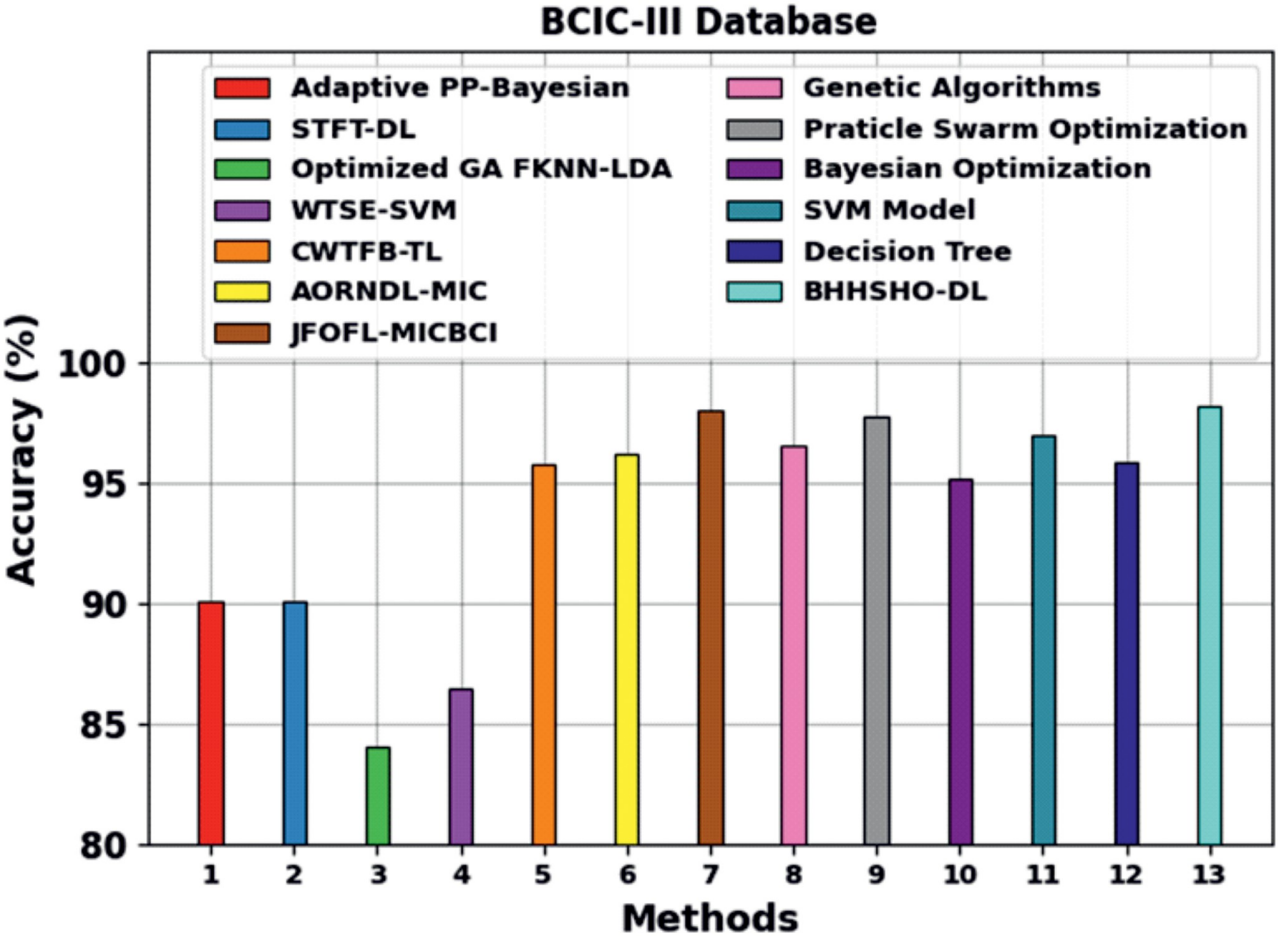
*Accu*_*y*_ outcome of the BHHSHO-DL model with BCIC-III database.

Table 2 and Fig 11 provide a detailed comparison study of the BHHSHO-DL technique using the BCIC-III database [32–34]. These experiments show that the Adaptive PP-Bayesian, STFT-DL, optimized GA FKNN-LDA, and WTSE-SVM models have reached ineffectual outcomes. The CWTFB-TL, AORNDL-MIC, JFOFL-MICBCI, Genetic Algorithm (GA), Particle Swarm Optimization (PSO), Bayesian Optimization (BO), SVM, and Decision Tree (DT) techniques have also gained adjacent performance. However, the BHHSHO-DL approach reaches optimal performance with greater performance and an *accu*_*y*_ of 98.15%.

**Table 2:** *Accu*_*y*_ outcome of BHHSHO-DL method under BCIC-III database compared with other models [32–3,4].

BCIC-III Database	
Methods	*Accu*_*y*_
Adaptive PP-Bayesian	90.04
STFT-DL	90.04
Optimized GA FKNN-LDA	84.04
WTSE-SVM	86.44
CWTFB-TL	95.75
AORNDL-MIC	96.18
JFOFL-MICBCI	97.95
GA	96.53
PSO	97.71
BO	95.11
SVM	96.98
DT	95.87
BHHSHO-DL	98.15

**Fig 12:**
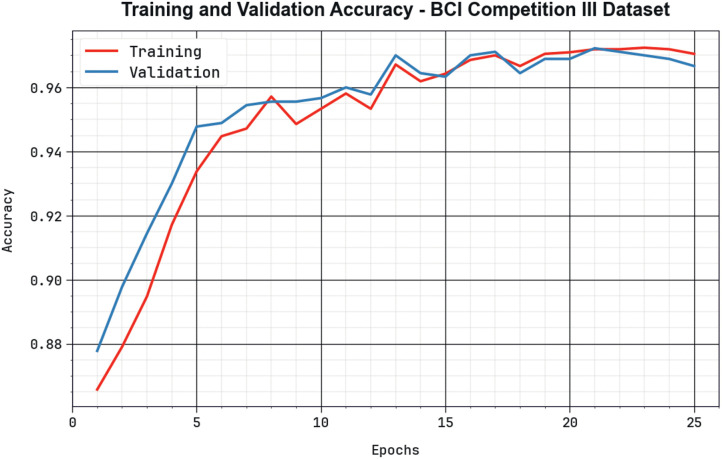
*Accu*_*y*_ curve of the BHHSHO-DL method with BCIC-III database.

The effectiveness of the BHHSHO-DL approach with the BCIC-III database is presented in Fig 12 in the method of training accuracy (TRAA) and validation accuracy (VALA) outcomes. This outcome displays a valuable analysis of the behaviour from the BHHSHO-DL approach in numerous epochs, indicating its learning method and generalizability. Mainly, the outcome infers a consistent enhancement from the TRAA and VALA with enhanced epoch counts. It guarantees the adaptive nature of the BHHSHO-DL methodology in pattern detection development on both datasets. The increased tendency in VALA outlines the capability of the BHHSHO-DL methodology to change to the TRA data and excel in offering a precise classifier of unobserved information, pointing out strong generalizability.

**Fig 13:**
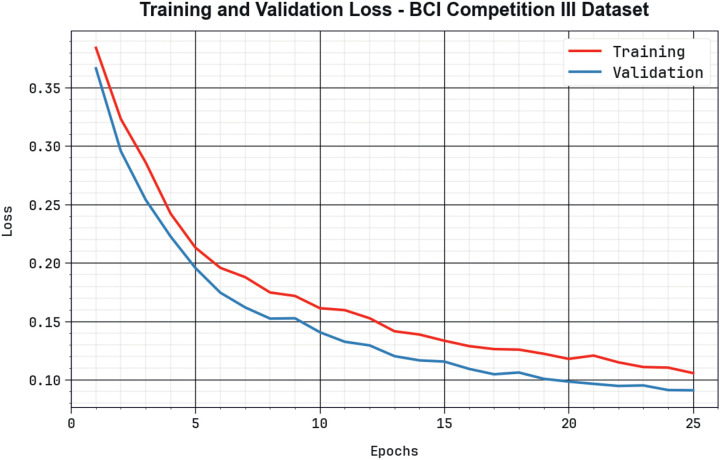
Loss curve of the BHHSHO-DL method with BCIC-III database.

Fig 13 demonstrates the training loss (TRLA) and validation loss (VALL) results of the BHHSHO-DL technique with the BCIC-III dataset over different epoch counts. The advanced minimum in TRLA highlights the BHHSHO-DL technique, enhancing the weights and decreasing the classification error on both datasets. The outcome denotes a well-defined data set of the BHHSHO-DL approach associated with the TRA data, underlining its ability to capture patterns. The BHHSHO-DL approach constantly increases its parameters to reduce the variances between the real and predictive TRA classes.

**Fig 14:**
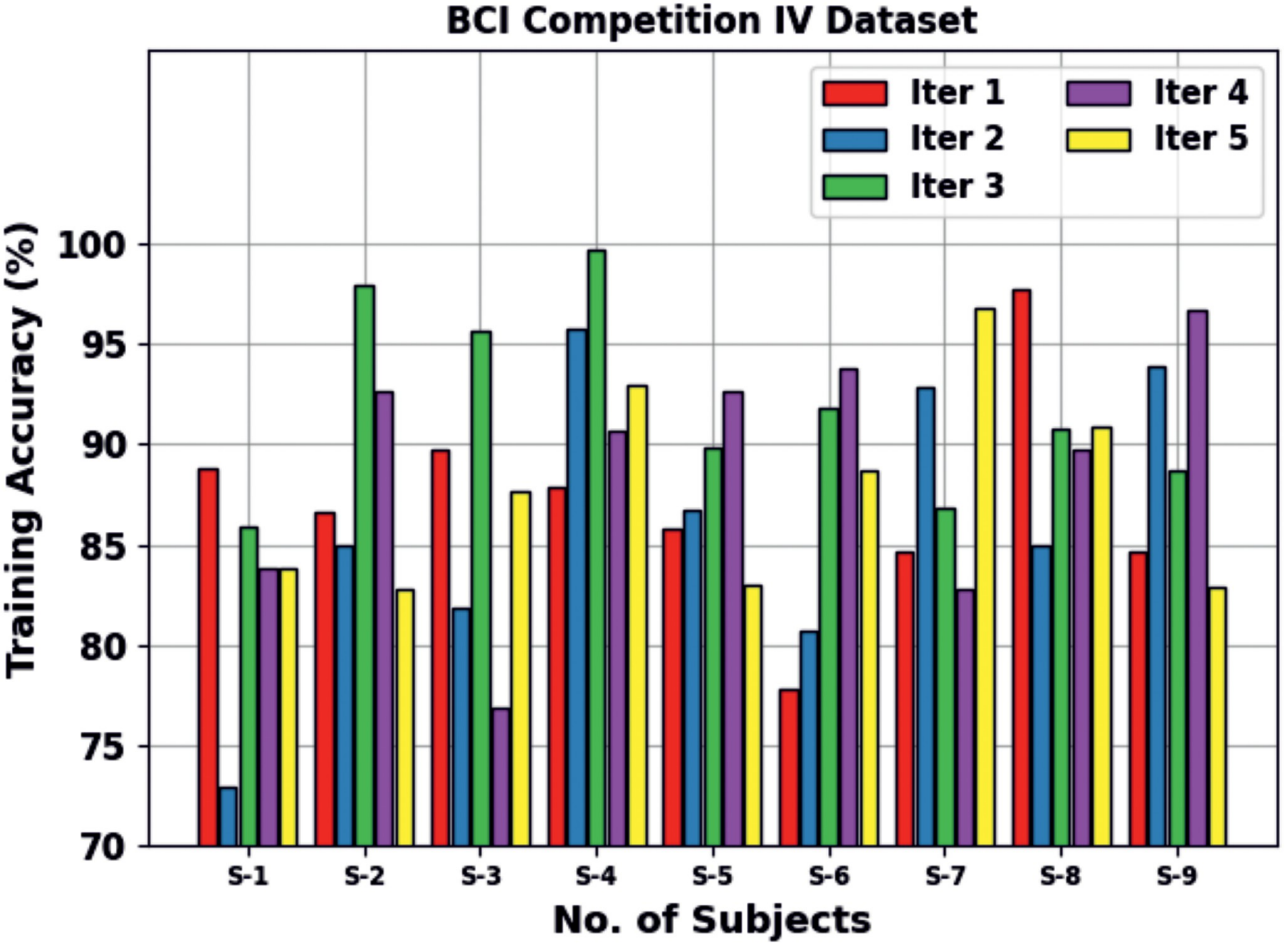
Training accuracy outcome of BHHSHO-DL model with BCIC-IV database.

A detailed training accuracy outcome is offered by the BHHSHO-DL method with the BCIC-IV database and illustrated in Table 3 and Fig 14. These stimulation outcomes indicate that the BHHSHO-DL technique provides increased performance at several runs. It is perceived that the BHHSHO-DL technique gains average S-1 of 83.05%, S-2 of 88.99%, S-3 of 86.35%, S-4 of 93.41%, S-5 of 87.59%, S-6 of 86.55%, S-7 of 88.78%, S-8 of 90.82%, and S-9 of 89.39%, respectively.

**Table 3:** Training accuracy result of the BHHSHO-DL method under the BCIC-IV database.

BCIC-IV Database										
No. of runs	S1	S2	S3	S4	S5	S6	S7	S8	S9	Average
Iter 1	88.80	86.67	89.70	87.88	85.77	77.76	84.66	97.80	84.65	**87.08**
Iter 2	72.86	84.93	81.87	95.81	86.69	80.67	92.85	84.96	93.94	**86.06**
Iter 3	85.90	97.91	95.70	99.71	89.88	91.80	86.79	90.75	88.67	**91.90**
Iter 4	83.85	92.68	76.81	90.69	92.68	93.83	82.81	89.75	96.76	**88.87**
Iter 5	83.83	82.75	87.69	92.96	82.93	88.68	96.79	90.84	82.91	**87.71**
**Average**	**83.05**	**88.99**	**86.35**	**93.41**	**87.59**	**86.55**	**88.78**	**90.82**	**89.39**	**88.32**

**Fig 15:**
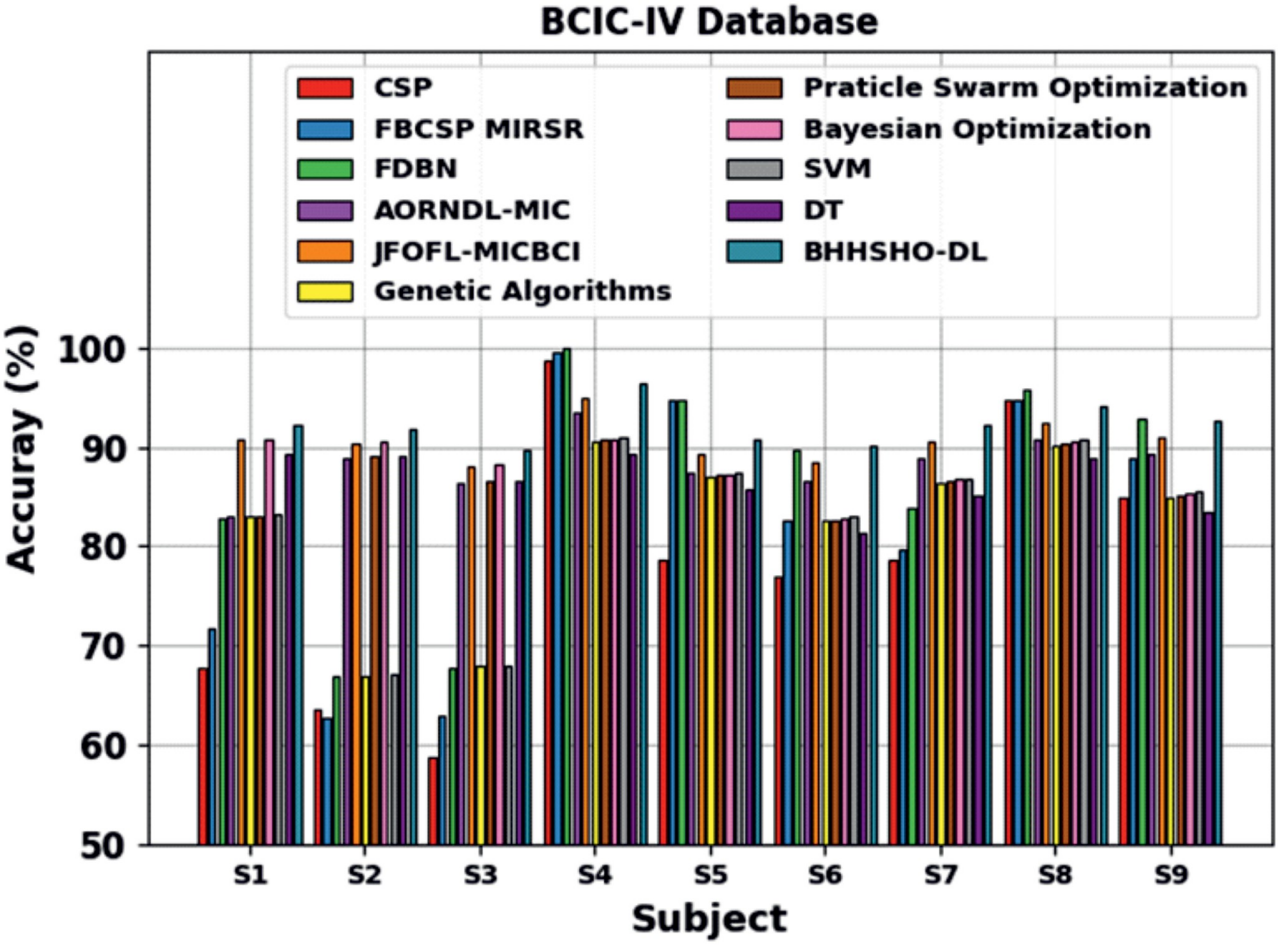
*Accu*_*y*_ outcome of the BHHSHO-DL methodology at BCIC-IV database.

An extensive comparative outcome of the BHHSHO-DL method with the BCIC-IV database is described in Table 4 and Fig 15. The performance outcomes display that the CSP and FBCSP MIRSR techniques have obtained poorer outcomes. Similarly, the FDBN, AORNDL-MIC, and JFOFL-MICBCI methods have obtained remarkable performance. Likewise, GA, PSO, BO, SVM and DT models attained slightly higher values. The BHHSHO method illustrates a notable merit in balancing exploration and exploitation compared to conventional GA and PSO. This capability is significant for effectually navigating complex search spaces and finding optimal solutions, as shown by its superior convergence properties emphasized in Figs 5–7. The BHHSHO’s refined approach to optimization allows it to adapt more dynamically to the data landscape, resulting in improved performance metrics across diverse datasets comprising the BCIC-IV and BCIC-III. Also, the BHHSHO-DL technique presents superior performance on higher *accu*_*y*_ values with S-1 of 92.16%, S-2 of 91.86%, S-3 of 89.61%, S-4 of 96.47%, S-5 of 90.77%, S-6 of 90.10%, S-7 of 92.30%, S-8 of 94.14%, and S-9 of 92.62%, correspondingly.

**Table 4:** *Accu*_*y*_ outcome of BHHSHO-DL methodology under BCIC-IV database compared with other models [32–3,4].

BCIC-IV Database											
Subject	CSP	FBCSP MIRSR	FDBN	AORNDL-MIC	JFOFL-MICBCI	GA	PSO	BO	SVM	DT	BHHSHO-DL
S1	67.77	71.61	82.81	82.95	90.69	82.96	83.10	90.86	83.12	89.22	92.16
S2	63.58	62.73	66.82	88.84	90.41	67.00	89.00	90.59	67.18	89.07	91.86
S3	58.66	62.84	67.68	86.38	88.05	67.85	86.55	88.22	68.03	86.57	89.61
S4	98.63	99.57	99.87	93.42	94.97	90.50	90.68	90.83	90.99	89.29	96.47
S5	78.69	94.73	94.77	87.50	89.25	86.94	87.12	87.29	87.46	85.68	90.77
S6	76.87	82.63	89.62	86.64	88.45	82.52	82.69	82.84	83.01	81.24	90.10
S7	78.60	79.67	83.82	88.86	90.56	86.39	86.55	86.72	86.88	85.05	92.30
S8	94.76	94.68	95.84	90.75	92.51	90.22	90.40	90.55	90.72	88.89	94.14
S9	84.80	88.83	92.85	89.19	90.90	84.97	85.13	85.29	85.45	83.49	92.62
**Average**	**78.04**	**81.92**	**86.01**	**88.28**	**90.64**	**82.15**	**86.80**	**88.13**	**82.54**	**86.50**	**92.23**

**Fig 16:**
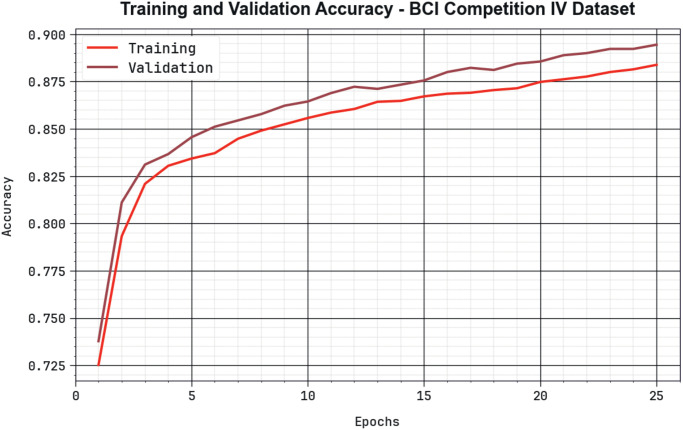
*Accu*_*y*_ curve of the BHHSHO-DL model at BCIC-IV database.

The effectiveness of the BHHSHO-DL model BCIC-IV database is portrayed in Fig 16 in the procedure of TRAA and VALA curves. The outcome shows a beneficial explanation of the behaviour of the BHHSHO-DL technique at unpredictable epochs, demonstrating its learning development and generalizability. Noticeably, the outcome gathers a continuous enhancement in the TRAA and VALA with development in epoch counts. It provides the adaptive nature of the BHHSHO-DL technique in the pattern detection method with two data. The improved tendency in VALA outlines the ability of the BHHSHO-DL technique to modify the TRA data and excel in presenting a precise classifier of hidden data, showing strong generalizability.

**Fig 17:**
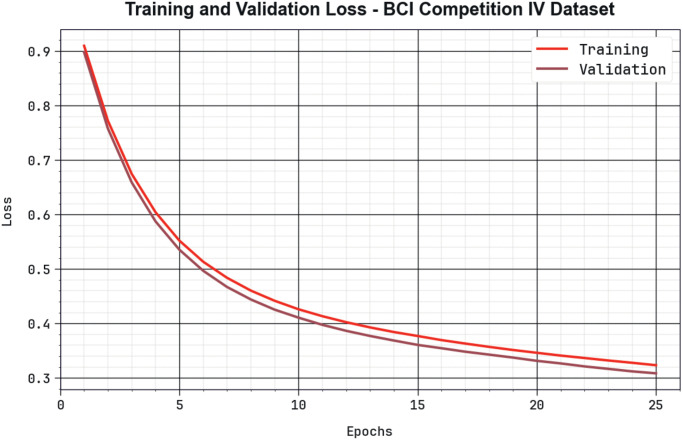
Loss curve of BHHSHO-DL approach with BCIC-IV database.

Fig 17 displays a complete view of the TRLA and VALL outcomes of the BHHSHO-DL method with the BCIC-IV dataset across different epoch counts. The gradual reduction in TRLA emphasizes the BHHSHO-DL method, optimizing the weights and minimizing the classification error on both data. The outcome exposes evident data of the BHHSHO-DL method relevant to the TRA data, underlining its ability to capture patterns from both databases. The BHHSHO-DL approach constantly increases its parameters to reduce the variances among the predictive and real TRA classes. Hence, the BHHSHO-DL approach is used to enhance the MI classification process.

## 5. Conclusion

This article presents a unique BHHSHO-DL technique-based MI classification for BCI. The BHHSHO-DL technique mainly exploits the hyperparameter-tuned DL approach for identifying MI for BCI. To obtain this, the BHHSHO-DL method followed four main methods: WPD-based preprocessing, DenseNet-based feature extractor, BHHSHO-based parameter tuning, and CAE-based classification. At this stage, the enhanced DenseNet technique extracts the complex and hierarchical feature patterns from the data preprocessing. Moreover, the improved DenseNet model extracts the complex and hierarchical feature patterns from the data preprocessing. Meanwhile, the BHHSHO technique-based hyperparameter tuning process is performed to elect optimal parameter values of the enhanced DenseNet approach. Finally, the classification model is implemented using the CAE model. The experimental evaluation of the BHHSHO-DL technique is accomplished on a benchmark database. The performance validation of the BHHSHO-DL methodology portrayed a superior accuracy value of 98.15% and 92.23% over other techniques under BCIC-III and BCIC-IV datasets. The existing methods for MI classification face limitations encompassing high computational complexity, difficulties in generalizing across various datasets, and threats with feature redundancy and overfitting. Some methods need more efficient preprocessing, affecting accuracy and robustness, while others need help with precision loss or dataset variability. Future research must develop more effectual approaches that balance computational demands with accuracy, enhance generalization through advanced feature extraction and data augmentation methods, and improve preprocessing methodologies to handle noisy data more effectively. Moreover, exploring novel hybrid optimization models and TL methods could address existing threats and enhance the model’s overall performance.

## References

[1] Mohammadi E.; Daneshmand P.G. and Khorzooghi S.M.S.M. Electroencephalography-based brain–Computer interface motor imagery classification. Journal of Medical Signals and Sensors, 2022, 12(1), p.40. doi: 10.4103/jmss.JMSS_74_20 35265464 PMC8804596

[2] Sun B.; Wu Z.; Hu Y. and Li T. Golden subject is everyone: A subject transfer neural network for motor imagery-based brain computer interfaces. Neural Networks, 2022, 151, pp.111–120. doi: 10.1016/j.neunet.2022.03.025 35405471

[3] Khanam T.; Siuly S. and Wang H. An optimized artificial intelligence based technique for identifying motor imagery from EEGs for advanced brain computer interface technology. Neural Computing and Applications, 2023, 35(9), pp.6623–6634.

[4] Ma J.; Yang B.; Qiu W.; Li Y.; Gao S. and Xia X. A large EEG dataset for studying cross-session variability in motor imagery brain-computer interface. Scientific Data, 2022, 9(1), p.531. doi: 10.1038/s41597-022-01647-1 36050394 PMC9436944

[5] Narayanan V., Nithya P. and Sathya M., 2023. Effective lung cancer detection using deep learning network. *Journal of Cognitive Human-Computer Interaction*, (2), pp.15–5.

[6] Wang, X., Yang, R., Huang, M., Yang, Z. and Wan, Z., 2021, March. A hybrid transfer learning approach for motor imagery classification in brain-computer interface. In *2021 IEEE 3rd Global Conference on Life Sciences and Technologies (LifeTech)* (pp. 496–500). IEEE.

[7] Tong J.; Xing Z.; Wei X.; Yue C.; Dong E.; Du S.; et al. Towards Improving Motor Imagery Brain–Computer Interface Using Multimodal Speech Imagery. Journal of Medical and Biological Engineering, 2023, 1–11.37363129

[8] Arpaia, P.; Esposito, A.; Moccaldi, N.; Natalizio, A. and Parvis, M. Online processing for motor imagery-based brain-computer interfaces relying on EEG. In 2023 IEEE International Instrumentation and Measurement Technology Conference (I2MTC) 2023, 01–06.

[9] Sadiq M.T.; Yu X.; Yuan Z.; Aziz M.Z.; Siuly S. and Ding W. Toward the development of versatile brain–computer interfaces. IEEE Transactions on Artificial Intelligence, 2021, 2(4), 314–328.

[10] Dumitrescu C.; Costea I.-M.; Semenescu A. Using Brain-Computer Interface to Control a Virtual Drone Using Non-Invasive Motor Imagery and Machine Learning. Applied Sciences, 2021, 11, 11876.

[11] Zheng M. and Lin Y., 2024. A deep transfer learning network with two classifiers based on sample selection for motor imagery brain-computer interface. *Biomedical Signal Processing and Control*, 89, p.105786.

[12] Medhi K., Hoque N., Dutta S.K. and Hussain M.I., 2022. An efficient EEG signal classification technique for Brain–Computer Interface using hybrid Deep Learning. *Biomedical Signal Processing and Control*, 78, p.104005.

[13] Cano-Izquierdo J.M., Ibarrola J. and Almonacid M., 2023. Applying deep learning in brain computer interface to classify motor imagery. *Journal of Intelligent & Fuzzy Systems*, (Preprint), pp.1–14.

[14] Yu S., Wang Z., Wang F., Chen K., Yao D., Xu P., et al. 2024. Multiclass classification of motor imagery tasks based on multi-branch convolutional neural network and temporal convolutional network model. *Cerebral Cortex*, 34(2), p.bhad511. doi: 10.1093/cercor/bhad511 38183186

[15] Malibari A.A., Al-Wesabi F.N., Obayya M., Alkhonaini M.A., Hamza M.A., Motwakel A., et al. 2022. Arithmetic optimization with retinanet model for motor imagery classification on brain computer interface. *Journal of healthcare engineering*, 2022. doi: 10.1155/2022/3987494 35368960 PMC8970805

[16] Echtioui A., Zouch W., Ghorbel M., Mhiri C. and Hamam H., 2023. Classification of BCI Multiclass Motor Imagery Task Based on Artificial Neural Network. *Clinical EEG and Neuroscience*, p.15500594221148285. doi: 10.1177/15500594221148285 36604821

[17] Wang X., Hersche M., Magno M. and Benini L., 2024. MI-BMInet: An efficient convolutional neural network for motor imagery brain–Machine interfaces with EEG channel selection. *IEEE Sensors Journal*.

[18] Chu C., Xiao Q., Chang L., Shen J., Zhang N., Du Y., et al. 2023. EEG temporal information-based 1-D convolutional neural network for motor imagery classification. *Multimedia Tools and Applications*, 82(29), pp.45747–45767.

[19] Sharma R., Kim M. and Gupta A., 2022. Motor imagery classification in brain-machine interface with machine learning algorithms: Classical approach to multi-layer perceptron model. *Biomedical Signal Processing and Control*, 71, p.103101.

[20] Roy A.M., 2022. An efficient multiscale CNN model with intrinsic feature integration for motor imagery EEG subject classification in brain-machine interfaces. *Biomedical Signal Processing and Control*, 74, p.103496.

[21] Roy A.M., 2022. Adaptive transfer learning-based multiscale feature fused deep convolutional neural network for EEG MI multiclassification in brain–computer interface. *Engineering Applications of Artificial Intelligence*, 116, p.105347.

[22] Kumari A., Edla D.R., Reddy R.R., Jannu S., Vidyarthi A., Alkhayyat A. et al. 2024. EEG-based motor imagery channel selection and classification using hybrid optimization and two-tier deep learning. *Journal of Neuroscience Methods*, 409, p.110215. doi: 10.1016/j.jneumeth.2024.110215 38968976

[23] Xie Y. and Oniga S., 2023. Classification of motor imagery EEG signals based on data augmentation and convolutional neural networks. *Sensors*, 23(4), p.1932. doi: 10.3390/s23041932 36850530 PMC9961359

[24] Echtioui A., Zouch W., Ghorbel M., Mhiri C. and Hamam H., 2024. Classification of BCI multiclass motor imagery task based on artificial neural network. *Clinical EEG and Neuroscience*, 55(4), pp.455–464. doi: 10.1177/15500594221148285 36604821

[25] Alsuradi H., Khattak A., Fakhry A. and Eid M., 2024. Individual-finger motor imagery classification: a data-driven approach with Shapley-informed augmentation. *Journal of Neural Engineering*, 21(2), p.026013. doi: 10.1088/1741-2552/ad33b3 38479013

[26] Arı E. and Taçgın E., 2024. NF-EEG: A generalized CNN model for multi class EEG motor imagery classification without signal preprocessing for brain computer interfaces. *Biomedical Signal Processing and Control*, 92, p.106081.

[27] Huang J.S., Liu W.S., Yao B., Wang Z.X., Chen S.F. and Sun W.F., 2021. Electroencephalogram-Based Motor Imagery Classification Using Deep Residual Convolutional Networks. *Frontiers in Neuroscience*, 15. doi: 10.3389/fnins.2021.774857 34867174 PMC8635693

[28] Wang K., Jiang P., Meng J. and Jiang X., 2022. Attention-based DenseNet for pneumonia classification. *IRBM*, 43(5), pp.479–485.

[29] Stateczny A., Praveena H.D., Krishnappa R.H., Chythanya K.R. and Babysarojam B.B., 2023. Optimized Deep Learning Model for Flood Detection Using Satellite Images. *Remote Sensing*, 15(20), p.5037.

[30] Chen M., Shi X., Zhang Y., Wu D. and Guizani M., 2017. Deep feature learning for medical image analysis with convolutional autoencoder neural network. *IEEE Transactions on Big Data*, 7(4), pp.750–758.

[31] Lemm S., Schafer C. and Curio G., 2004. BCI competition 2003-data set III: probabilistic modeling of sensorimotor/spl mu/rhythms for classification of imaginary hand movements. *IEEE Transactions on Biomedical Engineering*, 51(6), pp.1077–1080. 15188882 10.1109/TBME.2004.827076

[32] Yang E., Shankar K., Perumal E. and Seo C., 2023. Optimal Fuzzy Logic Enabled EEG Motor Imagery Classification for Brain Computer Interface. *IEEE Access*.

[33] Lin R., Dong C., Zhou P., Ma P., Ma S., Chen X., et al. 2024. Motor imagery EEG task recognition using a nonlinear Granger causality feature extraction and an improved Salp swarm feature selection. *Biomedical Signal Processing and Control*, 88, p.105626.

[34] Ganesh S., Kannadhasan S. and Jayachandran A., 2024. Multi class robust brain tumor with hybrid classification using DTA algorithm. *Heliyon*, 10(1). 10.1016/j.heliyon.2023.e23610 PMC10770571 38187263

